# Lipid lowering therapy in primary and secondary prevention in Austria: are LDL-C goals achieved? Results from the DA VINCI study

**DOI:** 10.1007/s00508-021-01978-w

**Published:** 2021-12-06

**Authors:** Peter Siostrzonek, Helmut Brath, Robert Zweiker, Heinz Drexel, Robert Hoelzl, Margit Hemetsberger, Kausik K. Ray

**Affiliations:** 1 Department of Internal Medicine 2, Cardiology, Ordensklinikum Linz, Barmherzige Schwestern, Seilerstätte 4, 4010 Linz, Austria; 2 Diabetes & Metabolic Outpatient Clinic, Health Centre Favoriten, Vienna, Austria; 3 Cardiology Department, Medical University Graz, Graz, Austria; 4 Vorarlberg Institute for Vascular Investigation and Treatment (VIVIT), Feldkirch, Austria; 5 Amgen GmbH, Vienna, Austria; 6 hemetsberger medical services, Vienna, Austria; 7 Imperial Centre for Cardiovascular Disease Prevention and Imperial Clinical Trials Unit, Imperial College London, London, UK

**Keywords:** Cross-sectional study, Guideline, Statin, PCSK9 inhibitor, Dyslipidemia

## Abstract

**Background:**

Cardiovascular disease (CVD) is the most frequent cause of death in Austria. The European Society of Cardiology (ESC)/European Atherosclerosis Society (EAS) guidelines recommend intensive lipid lowering therapy (LLT) in patients at high or very high CV risk. Lipid management and achievement of low-density lipoprotein cholesterol (LDL-C) goals in Austria have not recently been assessed.

**Methods:**

Subgroup analysis for Austria of a European 18 country, cross-sectional, observational study. Patients received LLT for primary (PP) or secondary prevention (SP). Data including LLT in the preceding 12 months and most recent LDL‑C were collected during a single visit between June 2017 and November 2018. Achievement of the risk-based 2016 and 2019 ESC/EAS LDL‑C goal while receiving stabilized LLT was assessed.

**Results:**

A total of 293 patients were enrolled from 8 Austrian sites, of which 200 (PP = 104, SP = 96) received stabilized LLT at the LDL‑C measurement date. Overall, 58% (71% PP, 43% SP) and 38% (52% PP, 23% SP) achieved the risk-based 2016 and 2019 goals, respectively. Most patients received moderate-intensity statin monotherapy (46%), while 34% used high-intensity statin monotherapy. Combination therapy of moderate/high-intensity statin with ezetimibe (12%), or proprotein convertase subtilisin/kexin type 9 (PCSK9) inhibitors with statin ± ezetimibe (1%), was used infrequently.

**Conclusion:**

The current Austrian routine lipid management using mainly moderate-intensity or high-intensity statin monotherapy is insufficient to attain ESC/EAS guideline goals, in particular the more stringent 2019 recommendations, a situation comparable to other participating European countries. In addition to switching to and optimizing doses of high-intensity statins, a combination with ezetimibe or PCSK9 inhibitors will be needed in many cases.

**Supplementary Information:**

The online version of this article (10.1007/s00508-021-01978-w) contains supplementary material, which is available to authorized users.

## Introduction

Cardiovascular (CV) events are the most frequent cause of death in Austria with 36.1% of all deaths occurring in 2020, corresponding to 32,663 lives lost in Austria alone. Women (39.1%, *n* = 17,908) are more frequently affected than men (33.0%, *n* = 14,755) [1]. Lifestyle, blood pressure and lipid levels are among the core modifiable CV risk factors addressed by the joint guidelines of the European Society of Cardiology (ESC) and the European Atherosclerosis Society (EAS) [2, 3]. There is increasing evidence [4–6] that lowering low density lipoprotein cholesterol (LDL-C) levels improves clinical benefit. This led to a further decrease in recommended LDL‑C goals for all patients, moderate to very high risk, in the 2019 edition of the ESC/EAS guidelines [3] compared to the 2016 edition [2]; however, attainment of guideline-recommended goals has often been demonstrated as being difficult to achieve in clinical practice. A study conducted in Austria in 2009/2010 found that attainment of the then recommended LDL‑C goal of <70 mg/dl was low and differed severely between Austrian federal states ranging from 5.9% to 38.5% [7]. Despite an increase in available treatment options, the most recent European Action on Secondary and Primary Prevention by Intervention to Reduce Events (EUROASPIRE V) survey [8] showed that as many as 71% of very high-risk patients did not achieve the 2016 ESC/EAS LDL‑C goals in the 27 participating countries; however, results varied widely across countries and even between centers. The Dyslipidemia International Study (DYSIS) II, investigating LDL‑C target attainment, lipid lowering therapy (LLT) usage, and CV outcomes in patients suffering from stable coronary heart disease (CHD) or acute coronary syndrome (ACS) in 17 countries in 2012–2013, showed >90% statin usage in CHD at enrolment at a mean dose of 25 ± 18 mg, but only 65.2% in ACS. The LDL‑C levels <70 mg/dL were only achieved in 29.4% of CHD and 18.9% of ACS patients. The authors mandated intensification of LLT in these very high-risk patients [9]. Austrian data are available from DYSIS I published in 2011 by Drexel et al. [10] and the results were comparable to the international data published later by Gitt et al. for DYSIS II [9]. Most efforts to estimate attainment of LDL‑C goals thus date from periods guided by older iterations of recognized international guidelines, therefore, there was a need to investigate the current situation with the aim to identify potential needs for improvement considering more stringent LDL‑C goals defined by the 2019 ESC/EAS dyslipidemia guidelines.

The aim of the present DA VINCI (EU-Wi**d**e Cross-Section**a**l Obser**v**atio**n**al Study of Lipid-Modifying Therapy Use in Se**c**ondary and Pr**i**mary Care) study was to assess how current clinical practice impacts LDL‑C goal attainment. Therefore, LLT usage for primary and secondary prevention of atherosclerotic cardiovascular disease (ASCVD) in Europe was comprehensively described; the Austrian cohort is presented here and descriptively compared to the previously published overall European DA VINCI findings [11].

## Patients, material and methods

Full details of the study methods are described in the overall DA VINCI study publication by Ray et al. [11]. In brief, this was an international cross-sectional study enrolling adults receiving LLT at primary and secondary care clinics across 18 European countries between 21 June 2017 and 20 November 2018. No formal study visits or study-related procedures were required. Data were collected from medical records at the enrolment visit using a standardized electronic case report form (eCRF) and included patient demographics and clinical characteristics; relevant past medical history, most recent lipid measurement recorded within 14 months prior to (and including) the enrolment visit, all LLT within 12 months before enrolment, history of intolerance to any statin at any dose, reason for LLT prescription in patients without previous ASCVD events and concomitant medications. Statin intensity was defined as per the American College of Cardiology/American Heart Association definition [12]. Primary prevention patients were assessed for the 10-year cardiovascular risk using the systematic coronary risk evaluation (SCORE) [13]. The SCORE was used to categorize primary prevention patients as low, moderate, high or very high risk. Secondary prevention patients with established ASCVD were categorized as very high risk by default. Estimated 10-year CV risk at LDL‑C measurement in these patients was estimated using the reduction of atherothrombosis for continued health (REACH) score [14]. The study schema in Fig. S1 of the online supplemental material shows relevant timepoints and measures.

The study included adult patients aged ≥18 years who were prescribed LLT and who had a documented LDL‑C measurement both within the timeframes defined above. There was an aim to enrol equal numbers of primary and secondary prevention patients on the site level. Secondary care sites aimed to enrol coronary, peripheral and cerebral (arterial) disease patients in a ratio of 1:2:2. Patients were excluded if they had a diagnosis of familial hypercholesterolemia with a history of CV events; further details are provided in Ray et al. [11].

### Aims and outcomes

The primary outcome was the percentage of patients achieving the LDL‑C goals recommended by the 2016 ESC/EAS guidelines while receiving stabilized LLT, which was defined as no change in dose or regimen for at least 28 days. Secondary outcomes included LLT use (type, dose, frequency; including combination therapy), assessed at the enrolment date and at the LDL‑C measurement date. As the study was completed before publication of the updated 2019 ESC/EAS guidelines, an exploratory post hoc analysis of the percentage of patients achieving the LDL‑C goals recommended in the 2019 guidelines was also conducted.

### Statistical analysis

All analyses were descriptive. Continuous variables are reported as mean and standard deviation (SD) or standard error (SE) for normally distributed data, and as median and 25th and 75th percentiles (Q1 and Q3, respectively) for data with a skewed distribution. For categorical variables, the number and percentage of patients in each category are reported.

## Results

### Study population

The Austrian study cohort included 293 patients, enrolled at 8 sites. The majority of patients were male (58.7%, *n* = 172) and of white ethnicity (94.9%, *n* = 278). The mean (SD) age was 68 (11) years. Of the patients, 142 were in primary prevention and 151 in secondary prevention. Fig. S2 shows the patient distribution by ASCVD status. Sixty percent (*n* = 175) were ever-smokers, 55% (*n* = 160) had been diagnosed with diabetes mellitus. Table 1 compares patient demographics and medical history of Austrian patients with the overall study population, revealing a slightly older study population in Austria, and a larger number of ever-smokers and patients with diabetes mellitus.

**Table 1:** Baseline demographics and clinical characteristics

	Austria *N* = 293	Overall [11] *N* = 5888
**Baseline demographics**		
*Male, n (%)*	172 (59)	3413 (58)
*Ethnicity, white, n (%)*	278 (95)	5435 (92)
*Age (years), mean (SD)*	68 (11)	65 (12)
*Systolic blood pressure (mm* *Hg), mean (SD)*	134.7 (18)	134.8 (17)
*Diastolic blood pressure (mm* *Hg), mean (SD)*	77.1 (11)	78.0 (11)
*BMI (kg/m*^*2*^*), mean (SD)*	28.8 (5)	28.7 (5)
*Smoking history, n (%)*		
Non-smoker	118 (40)	2854 (49)
Ex-smoker	134 (46)	2059 (35)
Light smoker	12 (4)	313 (5)
Moderate smoker	20 (7)	391 (7)
Heavy smoker	9 (3)	253 (4)
Missing	0 (0)	18 (<1)
*Diabetes mellitus*	160 (55)	2293 (39)
*Chronic kidney disease ≥grade 3, n (%)*	43 (15)	432 (7)
*Familial hypercholesterolemia, n (%)*	5 (2)	284 (5)
*Vascular bed involvement, n (%)*		
Coronary	52 (18)	1007 (17)
Cerebrovascular	67 (23)	1296 (22)
Peripheral	71 (24)	1125 (19)

*BMI* body mass index, *SD* standard deviation

### Cardiovascular risk profile

Primary prevention patients had a mean (SD) SCORE value of 2.4 (2.0), with 7.9% (*n* = 12) having low 10-year risk of fatal cardiovascular disease, 79.6% (*n* = 121) having moderate risk, 9.9% (*n* = 15) having high risk and 1.3% (*n* = 2) having very high risk. Compared to the overall population [11], the mean SCORE was slightly lower and a higher proportion of patients had moderate risk, whereas in the overall population the percentage of patients with high and very high cardiovascular risk was higher than in Austria (Table S2).

Secondary prevention patients had a mean (SD) REACH score of 40.0% (16.4%). Their predicted 10-year risk for a next fatal or non-fatal cardiovascular event was ≥0% to <10% in none of the patients, ≥10% to <20% in 5.1% (*n* = 7), ≥20% to <30% in 25.0% (*n* = 34), and ≥30% in 68.4% (*n* = 93). Compared to the overall population [11], mean REACH was slightly higher with a higher proportion of patients in the ≥30% group (Table S2).

### Lipid lowering therapy

**Fig. 1:**
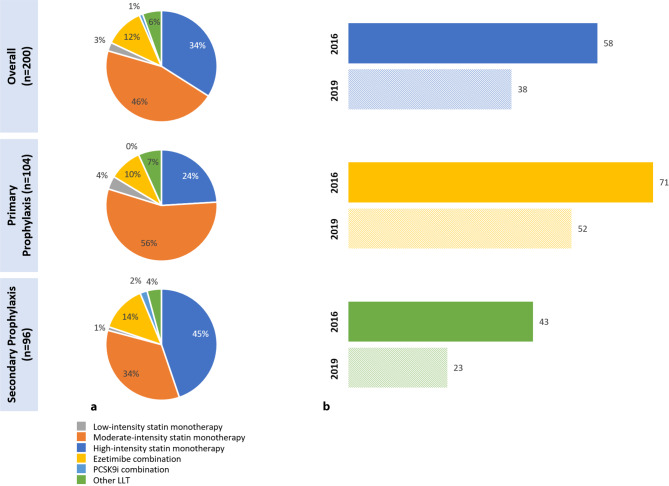
Stabilized lipid-lowering therapies and ESC/EAS goal attainment in Austria. **a** Lipid lowering therapy split (%). **b** Proportion of patients achieving risk-based goal (%). (LLT stabilized patients were defined as no change in dose or regimen for at least 28 days prior to LDL‑C measurement. Patients who were in secondary prevention at the visit date but whose first ASCVD event occurred after the date of their stabilized LDL‑C are categorized as primary prevention patients. 2016/2019 risk-based LDL‑C goals [2, 3]: a) low risk: 2016/2019, <116 mg/dL, b) moderate risk: 2016, <116 mg/dL; 2019, <100 mg/dL, c) high risk: 2016, <100 mg/dL; 2019, <70 mg/dL, d) very high risk: 2016, <70 mg/dL; 2019, <55 mg/dL). *EAS* European Atherosclerosis Society, *ESC* European Society of Cardiology, *LDL‑C* low density lipoprotein cholesterol, *LLT* lipid lowering therapy

As per inclusion criteria, all 293 patients received LLT, 200 patients were receiving stabilized LLT at the time of LDL‑C measurement and were evaluable for goal attainment: 95% (*n* = 190) received any statin, 34% (*n* = 68) a high-intensity statin monotherapy, 46% (*n* = 91) a moderate-intensity statin monotherapy and 3% (*n* = 5) a low intensity statin monotherapy; 12% received a moderate-high intensity statin-ezetimibe combination (*n* = 23) and 2 patients (1%) received a combination of proprotein convertase subtilisin/kexin type 9 inhibitors (PCSK9i) with statin and/or ezetimibe (Fig. 1; Table 2). Of the patients 93 were not on stabilized LLT at the time of LDL‑C measurement (*n* = 30 primary and *n* = 40 secondary prevention patients) or SCORE or estimated glomerular filtration rate (eGFR) was not available to determine the CV risk (*n* = 18 primary prevention patients) or they had other vascular secondary prevention (*n* = 5; Fig. S2). In the primary prevention setting (*n* = 104) 56% (*n* = 58) of individuals on stabilized LLT received moderate, 24% (*n* = 25) received high intensity statins alone and 10% (*n* = 10) received a statin-ezetimibe combination. In the secondary prevention setting (*n* = 96) 34% (*n* = 33) received moderate, 45% (*n* = 43) received high intensity statins alone, and 14% (*n* = 13) received statin-ezetimibe combination and 2% (*n* = 2) a combination with a PCSK9 inhibitor (Fig. 1). Compared to the overall study population [11], Austrian patients received similar treatments, except a slightly higher percentage receiving high intensity statins (Table 2).

**Table 2:** Use of lipid lowering therapy

Lipid lowering therapy, *n* (%)	LLT at enrolment^a^		Stabilized LLT^b^	
	Austria N = 293	Overall [11] N = 5888	Austria N = 200	Overall [11] N = 4112
**Any LLT**^c^				
*Any statin*	280 (96)	5554 (94)	190 (95)	3856 (94)
High intensity statin	143 (49)	2028 (34)	84 (42)	1306 (32)
Moderate intensity statin	126 (43)	3164 (54)	97 (49)	2279 (55)
Low intensity statin	6 (2)	226 (4)	5 (3)	171 (4)
Unknown intensity statin	5 (2)	136 (2)	4 (2)	100 (2)
*Ezetimibe*	43 (15)	667 (11)	25 (13)	491 (12)
*PCSK9i*	6 (2)	81 (1)	3 (2)	59 (1)
*Fibrates*	4 (1)	248 (4)	3 (2)	181 (4)
*Fish oils*	0 (0)	43 (<1)	0 (0)	36 (1)
**All LLT**^d^				
*Statin monotherapy*				
High intensity statin monotherapy	115 (39)	1787 (30)	68 (34)	1134 (28)
Moderate intensity statin monotherapy	115 (39)	2966 (50)	91 (45.5)	2131 (51.8)
Low intensity statin monotherapy	6 (2)	194 (3)	5 (2.5)	148 (3.6)
*Ezetimibe combination*	39 (13)	516 (9)	23 (11.5)	380 (9.2)
*PCSK9i combination*	3 (1)	64 (1)	2 (1.0)	49 (1.2)
*Other LLT*	15 (5)	361 (6)	11 (5.5)	270 (6.6)

Fibrates: bezafibrate, clofibrate, ciprofibrate, clofibride, clinofibrate, gemfibrozil, etofibrate, fenofibrate, ronifibrate, simfibrate

Statin intensity was defined per the American College of Cardiology/American Heart Association definition [12]

*LLT* lipid lowering therapy, *PCSK9i* proprotein convertase subtilisin/kexin type 9 (PCSK9) inhibitors

^a^Use of any LLT at the time of enrolment or any LLT prescribed in the 12 months before enrolment

^b^Stabilized LLT at LDL‑C measurement with no change in dose or regimen for at least 28 days prior to the LDL‑C measurement date

^c^Any use of a specific LLT regardless of whether a patient also received any other LLT

^d^All LLT used by each patient

### LDL-C levels and ESC/EAS goal attainment

Austrian patients with stabilized LLT had a mean (SE) LDL‑C level of 87.3 (2.3) mg/dL, 92.7 (2.9) mg/dL in primary and 81.3 (3.6) mg/dL in secondary prevention (Table S3), slightly lower than the overall study population. Among those evaluable for goal attainment, 58% achieved the risk-based 2016 ESC/EAS LDL‑C goal and 38% attained the 2019 ESC/EAS LDL‑C goal. In primary prevention goal achievement was 71% (2016) and 52% (2019), and lower in secondary prevention: 43% (2016) and 23% (2019) (Fig. 1). The goal attainment was slightly higher in Austria than the overall study population [11].

The 2016 ESC/EAS goal achievement was higher in patients with diabetes (61%, *n* = 67/109) versus those without diabetes (53%, *n* = 48/90) and in male (66%, *n* = 76/116) versus female patients (46%, *n* = 39/84). Presence versus absence of chronic kidney disease or age above versus lower than or equal to the median age of 69.0 years did not have an impact on the achievement of 2016 goals; however, patients without chronic kidney disease had a higher rate of 2019 goal attainment (41%, *n* = 65/158) compared to those affected by chronic kidney disease (26%, *n* = 11/42; Fig. S3).

## Discussion

In Austria, LDL‑C goal achievement has not been assessed recently [15–20] since the two large studies by Drexel et al. in 2011 [10] and Pichler et al. in 2013 [7], although relevant guidelines have been updated regularly. The last comprehensive analysis was a longitudinal, non-interventional study conducted in 2013 by Pichler et al. [7], where 45% were classified as very high and 55% as high cardiovascular risk patients according to the 2011 ESC/EAS guidelines. Individual LDL‑C goal values of <70 mg/dL were achieved by 14% while <100 mg/dl was attained by 61%. Vast differences were observed between the 9 federal states ranging from 38% (Carinthia) to 77% (Salzburg) for the <100 mg/dL goal and 6% (Carinthia) to 39% (Salzburg) for the <70 mg/dL goal.

The DA VINCI cross-sectional study of Austrian clinical practice was conducted while the 2016 ESC/EAS dyslipidemia guidelines were in use. The recommended goals were attained by only 58% of Austrian participants with a majority receiving moderate-intensity or high-intensity statins as a monotherapy. Goal attainment was higher in primary prevention (71%) compared to patients in secondary prevention (43%); however, after completion of the present study, updated ESC/EAS guidelines were issued in 2019, advocating more stringent LDL‑C goals. To assess the impact of these new recommendations on required adaptations for clinical practice, attainment of the new goals was estimated and was found to be low (38%), especially in secondary prevention (23%), strongly indicating that treatment needs to be intensified.

When comparing observations from the Austrian subgroup with the overall DA VINCI study population it was found that patients in Austria were slightly older (68 versus 65 years), and more patients had cardiovascular risk factors, such as diabetes (55% versus 39%), chronic kidney disease (15% versus 7%), or were current or former smokers (60% versus 51%). In primary prevention, fewer Austrian patients had high or very high risk compared to the overall study population (11% versus 21%). In secondary prevention, more patients had a REACH score of 30% or higher in Austria (68% versus 56%). Other patient characteristics were similar. The LLTs in Austria included slightly more frequent use of high-intensity statin monotherapy (34% versus 28%) and ezetimibe-statin combinations (12% versus 9%) in Austria compared to overall, but given the observed inadequate risk-based LDL‑C goal attainment in Austria and overall (58% versus 54%), LLT utilization of effective drugs or drug combinations must be considered insufficient. Attainment of the 2016 ESC/EAS goals in the DA VINCI study differed widely among participating countries, ranging between 21% in Ukraine and 73% in Italy [11], reflecting the diversity seen in EUROASPIRE V [8].

Austrian clinical practice at the time of the DA VINCI study (2017/2018) was guided by the 2016 ESC/EAS guidelines [2] and the 2016 iteration of the Austrian Lipid Consensus [21] along with Austrian reimbursement regulations. The 2016 Austrian Lipid Consensus advocated LDL‑C goals of <70 mg/dL and <100 mg/dL for very high-risk and high-risk patients, in accordance with the 2016 ESC/EAS guidelines [2]. For moderate and low-risk patients, however, higher LDL‑C goals of <130 mg/dL and <160 mg/dL, respectively, were adopted. It needs to be noted that the 2016 Austrian Lipid Consensus was published prior to the 2016 iteration of the ESC/EAS dyslipidemia guidelines and was thus still taking into consideration the 2011 ESC/EAS guidelines [22].

The fact that women were less likely treated to individual LDL-goal (Fig. S3) is of special interest and was observed also in other populations [23, 24]. It can be speculated that that the lower percentage of women reaching LDL goals could be part of the reason for the gender disparity in cardiovascular death, also disfavoring women [1].

The present study shows that most patients still receive statin monotherapy, with 13% receiving a combination with ezetimibe and only 1% receiving a PCSK9i combination. PCSK9i have been reimbursed in Austria since 2016, and only in secondary prevention patients [25]. This might explain the low use PCSK9i in the timeframe of data collection. Use of high-intensity statins and combination therapies was higher in secondary than primary prevention. Importantly, the use of low-intensity statins was limited to very few patients in secondary prevention. Recommended LDL‑C goals have been lowered with each subsequent iteration of the ESC/EAS guidelines, therefore intensification of therapy is required to attain these goals, as only 52% of primary and 23% of secondary prevention patients would receive the 2019 goals with their current treatment. Although the Austrian subpopulation of the DA VINCI study was too small and there was no appreciable diversity in prescribed treatment regimens to investigate differences in goal attainment between regimens, the overall study population encompassing nearly 6000 patients clearly showed notable trends towards higher goal achievement with intensification of treatment and combining different forms of LLT [11]. For the overall study population, it was estimated that among patients currently receiving moderate intensity statins approximately three quarters of high-risk and very high-risk primary prevention patients and half of secondary prevention would require at least double their current statin dose in order to achieve a 50% reduction in LDL‑C from their baseline LDL‑C; however, it was deemed unlikely by the authors that patients would achieve 2019 goals through increasing statin dosing alone.

DA VINCI showed that patients receiving statins in combination with non-statin add-ons, such as ezetimibe or PCSK9 inhibitors, were more likely to achieve 2019 goals overall and—most importantly—in patients with very high cardiovascular risk [11]. According to the 2019 ESC/EAS guidelines, an average LDL‑C reduction of approximately 85% could be expected with a combination of PCSK9i plus high intensity statin plus ezetimibe [3]. In secondary prevention, the REACH score to estimate risk of CV events found that 25% of ASCVD patients in the current study had a 10-year residual risk of ≥20% to <30% and 68.5% had ≥30% residual risk (mean [SD] 40.0% [16.4%]). According to the Cholesterol Treatment Trialists’ Collaborators (CTTC) meta-analyses [26] it can be estimated that in these patients with pre-existing ASCVD, reducing LDL‑C from the mean of 81.3 mg/dL to the 2019 ESC/EAS goal of below 55 mg/dL could lead to a 15% or higher relative reduction in CV events in 5 years, an absolute risk reduction for the REACH score of 6%, and a considerable associated mortality reduction.

Methodological strengths and limitations of the present study were discussed in detail in Ray et al. [11]. In brief, this study covered a broad range of care settings and patient groups, including previously less well-studied ones. The analysis of LDL‑C goal attainment as per the 2016 as well as the 2019 iterations of the ESC/EAS guidelines allowed to indicate possible paths towards achievement of the more stringent 2019 goals. Especially the comparison of the Austrian data presented here with the overall dataset allows an extrapolation of the impact of various treatment scenarios on goal attainment and thus requirements for adaptations in current practice; however, local prescribing regulations maintain strict limitations on the types of treatment reimbursed in specific patient scenarios and countries, and the positive impact of combinations therapies on goal attainment could only be demonstrated as a trend in low patient numbers in the extensive overall dataset.

## Conclusion

DA VINCI shows a large gap between 2016 ESC/EAS dyslipidaemia guideline recommendations and routine clinical practice in high and very high-risk ASCVD patients in Austria. The current routine management using mainly statin monotherapy of moderate or high intensity is inadequate, a situation comparable to other participating European countries. Especially the 2019 ESC/EAS LDL‑C goal for high-risk and very high-risk patients is largely unattainable on statin monotherapy. In addition to optimized high-intensity statins, a combination with non-statin LLT like ezetimibe or PCSK9 inhibitors will be needed to achieve guideline conform treatment in the majority of patients.

## Supplementary Information

Supplementary Appendix
