# Supplementary material for: Lipid lowering therapy in primary and secondary prevention in Austria: are LDL-C goals achieved? Results from the DA VINCI study
Source: Wien Klin Wochenschr. 2021 Dec 6;134(7-8):294–301. doi: 10.1007/s00508-021-01978-w (PMC9023395; doi:10.1007/s00508-021-01978-w)
Supplement: Supplementary file 1 — Supplementary Appendix [file 508_2021_1978_MOESM1_ESM.docx]

**Supplementary Appendix**

**Lipid lowering therapy in primary and secondary prevention in Austria: are LDL-C goals achieved? Results from the DA VINCI study**

**Figure S1: Depictions of the study schema**


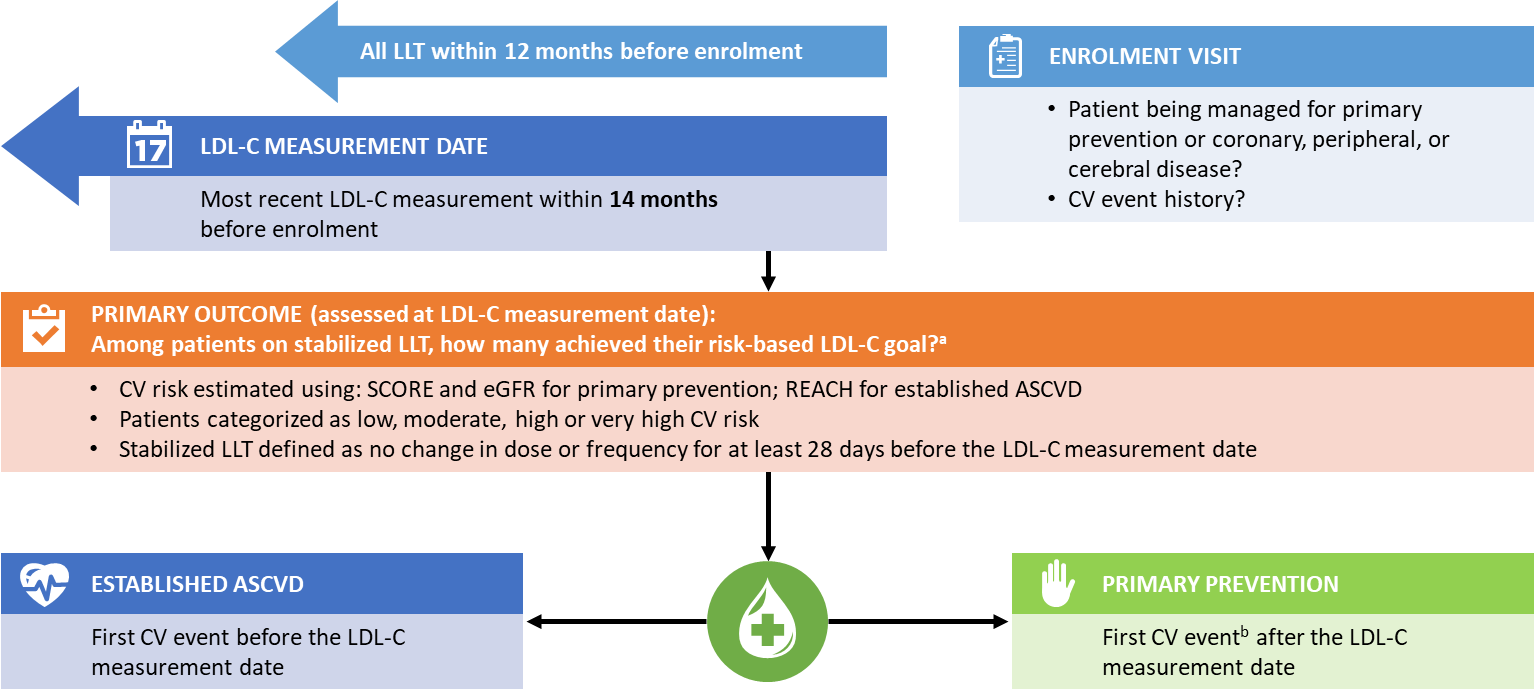


ASCVD = atherosclerotic cardiovascular disease events; CV = cardiovascular; eGFR = estimate glomerular filtrate; GFR = glomerular filtration rate; LDL-C = low density lipoprotein cholesterol; LLT = lipid-lowering therapy; PP = primary prevention; REACH = Reduction of Atherothrombosis for Continued Health; SCORE = Systematic Coronary Risk Evaluation.

Patients who were not stabilized on any LLT or had their LDL-C measurement taken before any LLT was initiated were not included in the assessment of the primary outcome. Includes patients enrolled as PP with no CV events.

^a^Patients enroled as secondary prevention who were not being managed for peripheral, vascular or coronary disease and who had other evidence of atherosclerosis or other manifestations of vascular disease at enrolment. ^b^Among patients considered as secondary prevention at the enrolment visit, 142 had their first cardiovascular event recorded after their most recent LDL-C measurement, hence they were analysed as primary prevention patients for outcomes assessed at LDL-C measurement, such as goal attainment. For outcomes assessed at enrolment, these 142 patients were analysed as secondary prevention.

Reproduced from Ray KK, et al. Eur J Prev Cardiol 2020 (1)

**Figure S2: Patient** **distribution by ASCVD status**


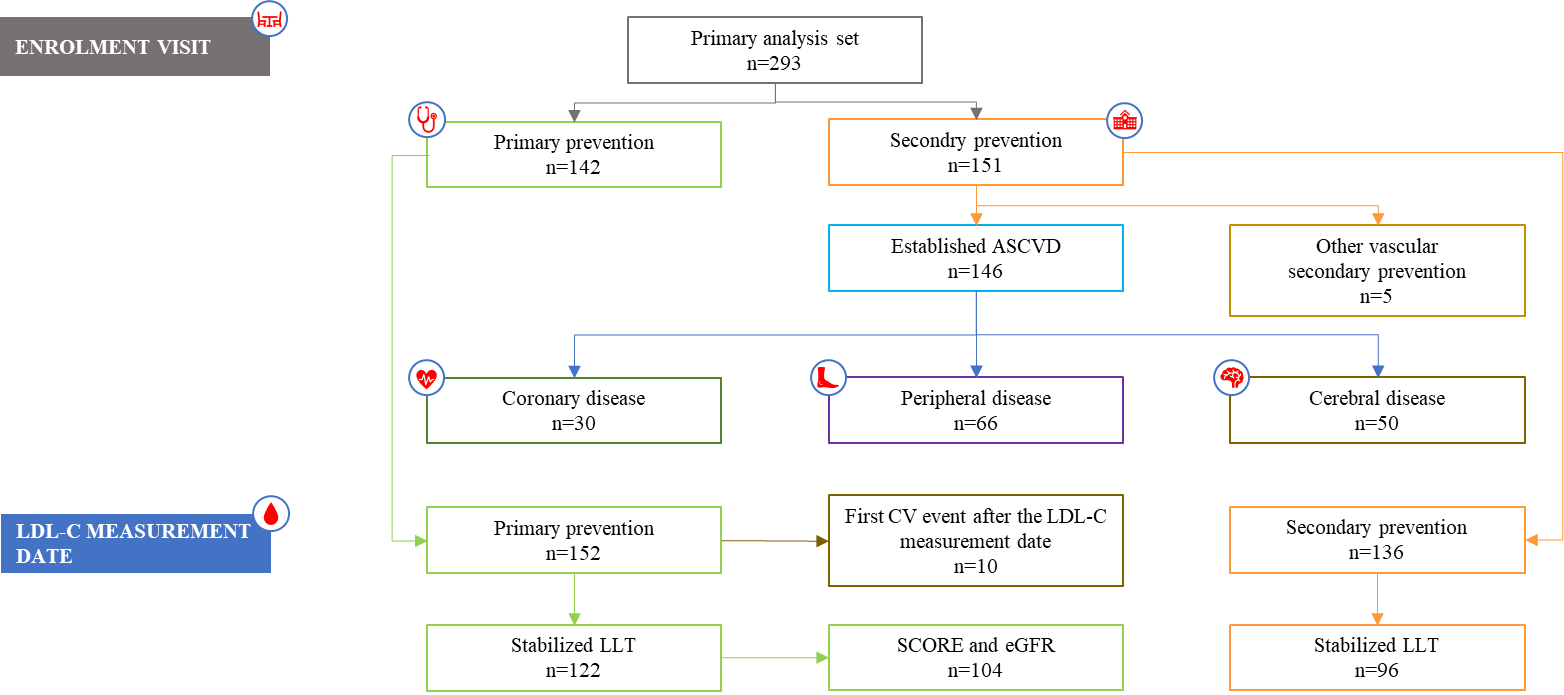


ASCVD, atherosclerotic cardiovascular disease; CV, cardiovascular; eGFR, estimated glomerular filtration rate; GFR, glomerular filtration rate; LDL-C, low-density lipoprotein cholesterol; LLT, lipid-lowering therapy; SCORE, Systematic Coronary Risk Evaluation

**Figure S3. Patients achieving risk-based goal, by diabetes status**, **sex, status of chronic kidney disease, and age** (%)


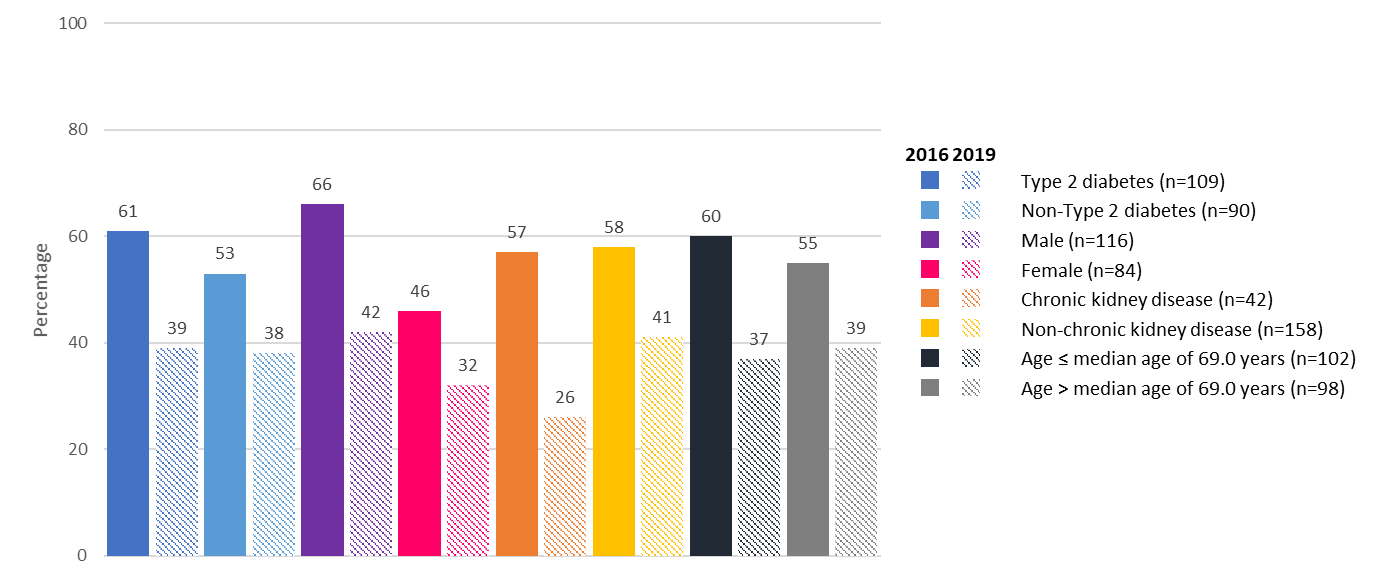


**Table S1. Enrolment, principal investigators, and site names, Austrian cohort**

| **Austria, N=293** | **Principal investigator** |
| --- | --- |
| Health Centre Favoriten, Vienna | Helmut Brath |
| Medical University Graz | Robert Zweiker |
| VIVIT Institute, Feldkirch | Heinz Drexel |
| Ordensklinikum Linz, Hospital Barmherzige Schwestern | Peter Siostrzonek |
| General Practitioner, Straden | Gudrun Zweiker |
| Medical University of Vienna, Dept of Medicine II | Klaus Distelmaier |
| Medical University of Vienna, Dept of Medicine III | Thomas M. Stulnig |
| Medical University Innsbruck | Christoph Ebenbichler |

Overall number of enrolled patients, N=5888

**Table S2. Predicted 10-year cardiovascular risk in primary and secondary prevention settings**

|  | **Austria (N = 293)** | **Overall (N = 5888)** |
| --- | --- | --- |
| **SCORE^a^: Primary prevention patients - N** | **152** | **3142** |
| n^b^ | 150 | 3019 |
| Mean (SD) | 2.4 (2.0) | 3.2 (2.8) |
| Low-risk, n (%) | 12 (7.9) | 285 (9.1) |
| Moderate-risk, n (%) | 121 (79.6) | 2077 (66.1) |
| High risk, n (%) | 15 (9.9) | 559 (17.8) |
| Very high-risk, n (%) | 2 (1.3) | 98 (3.1) |
| **REACH^c^: Secondary prevention patients - N** | **136** | **2659** |
| n^b^ | 134 | 2480 |
| Mean (SD), % | 40.0 (16.4) | 36.1 (15.2) |
| ≥0 to <10%, n (%) | 0 (0.0) | 7 (0.3) |
| ≥10 to <20%, n (%) | 7 (5.1) | 285 (10.7) |
| ≥20 to <30%, n (%) | 34 (25.0) | 703 (26.4) |
| ≥30%, n (%) | 93 (68.4) | 1485 (55.8) |

REACH, Reduction of Atherothrombosis for Continued Health (2); SCORE, Systematic Coronary Risk Evaluation (3); SD, standard deviation

SCORE low risk = 0, moderate risk = 1-4, high risk = 5-9, very high risk = ≥10

^a^ Ten-year risk of fatal cardiovascular disease.

^b^ For some patients SCORE or REACH could not be calculated; percentages are based on those patients where the risk score could be calculated as indicated by the lower-case n

^c^ 10-year risk for next cardiovascular event.

Estimated 10-year cardiovascular risk at low-density lipoprotein cholesterol measurement in primary prevention group and estimated 10-year risk of fatal and non-fatal cardiovascular events at low-density lipoprotein cholesterol measurement in established atherosclerotic cardiovascular disease group. Primary prevention: Data shown are for all patients considered primary prevention at low-density lipoprotein cholesterol measurement (Austria: n = 152; Overall: n=3142); of these, 122 and 2073, respectively, were on stabilized lipid-lowering therapy at low-density lipoprotein cholesterol measurement and had data available to calculate systematic coronary risk evaluation and glomerular filtration rate risk. Secondary prevention: Data shown are for all patients considered having established atherosclerotic cardiovascular disease at low-density lipoprotein cholesterol measurement (Austria: n=136; Overall: n=2659); of these, 96 and 2039, respectively, were on stabilized lipid-lowering therapy at low-density lipoprotein cholesterol measurement.

**Table S3. Baseline LDL-C levels in LLT stabilised patients, overall and by ASCVD status**

| **Mean (SE) LDL-C level, mg/dL** | **Austria (N = 293)** | **Overall (N = 5888)** |
| --- | --- | --- |
| Overall | 87.3 (2.3) n=218 | 91.4 (0.5) n=4668 |
| Primary prevention | 92.7 (2.9) n=118 | 98.0 (0.7) n=2558 |
| Secondary prevention | 81.3 (3.6) n=96 | 83.1 (0.8) n=2039 |

ASCVD, atherosclerotic cardiovascular disease; LLT, lipid/lowering therapy

Data from the overall study population are adapted from (4).

**References**

1. Ray KK, Molemans B, Schoonen WM, Giovas P, Bray S, Kiru G, et al. EU-Wide Cross-Sectional Observational Study of Lipid-Modifying Therapy Use in Secondary and Primary Care: the DA VINCI study. European Journal of Preventive Cardiology. 2020.

2. Wilson PW, D'Agostino R, Sr., Bhatt DL, Eagle K, Pencina MJ, Smith SC, et al. An international model to predict recurrent cardiovascular disease. The American journal of medicine. 2012;125(7):695-703.e1.

3. Conroy RM, Pyörälä K, Fitzgerald AP, Sans S, Menotti A, De Backer G, et al. Estimation of ten-year risk of fatal cardiovascular disease in Europe: the SCORE project. Eur Heart J. 2003;24(11):987-1003.

4. Ltd. A. EU-Wide Cross-Sectional Observational Study of Lipid-Modifying Therapy Use in Secondary and Primary Care (DA VINCI). Observational Research Study Report Abstract: 20150333. 2019 [Available from: <http://www.encepp.eu/encepp/openAttachment/studyResult/31477;jsessionid=EBpY9fN2-dt9mDy6_jQO7NEF1njLGiUMnhbS-L5KcCYV7f0kj_dv!953644876>.
